# High-Frequency Sonication as an Unconventional Solution to Control Fluid Loss in Water-Based Drilling Muds

**DOI:** 10.1021/acsomega.5c05870

**Published:** 2025-11-13

**Authors:** Anoop Kanjirakat, Arnel Carvero, Jocin James Abraham, Laith Abughaush, Mahmood Amani

**Affiliations:** † Department of Aeronautical and Automobile Engineering, 125853 Manipal Institute of Technology (MIT), Manipal Academy of Higher Education (MAHE), Manipal 576104, India; ‡ Petroleum Engineering Program, 146313 Texas A&M University at Qatar, Education City, P.O. Box 23874, Doha, Qatar

## Abstract

Conventional methods of mitigating fluid loss in drilling operations rely heavily on chemical additives and nanoparticles, which may alter mud properties and introduce environmental and cost challenges. While the technique of ultrasonication is widely used in the oil and gas industry, especially for mixing these additives and nanoparticles into mud samples, the direct impact of ultrasonication of the base drilling mud sample alone on fluid loss behavior has been largely overlooked. This study investigates the effectiveness of ultrasonication in modifying the filtrate loss characteristics of water-based mud without any fluid loss additives. Drilling mud samples were subjected to ultrasonic treatment and compared with unsonicated samples through a series of high-pressure high-temperature (HPHT) filtration tests using ceramic disks with average pore throat sizes of 5–35 μm. The results demonstrate that sonicated mud significantly reduces filtrate loss, particularly in disks with smaller pore sizes, compared with unsonicated mud. Reductions in filtrate loss of approximately 16–45% were achieved with the 5, 10, and 20 μm pore size disks when sonicated mud was tested. These improvements were associated with the thinner and less permeable (0.2–0.4 mm) filter cakes compared to those of unsonicated mud, contributing to improved fluid retention. However, at a pore throat size of 35 μm, a significant increase in fluid loss was observed, suggesting that the benefits of ultrasonication are limited when the pore sizes exceed the effective range of the modified particle size distribution in the mud. Particle size distribution and scanning electron microscopy (SEM) data confirmed that ultrasonication fragmented and dispersed larger particles/aggregations, leading to a more stable suspension in the mud and denser mud cakes. The findings from this study demonstrate that ultrasonication of the base drilling mud alone is sufficient to improve its filtrate loss properties in most cases without the need for loss additives while being environmentally friendly and cost-effective. However, the study also implies the importance of adapting mud systems to formation properties, highlighting the critical role of the interaction between the particle size and pore dimensions in influencing fluid loss behavior and mud cake properties.

## Graphical abstract

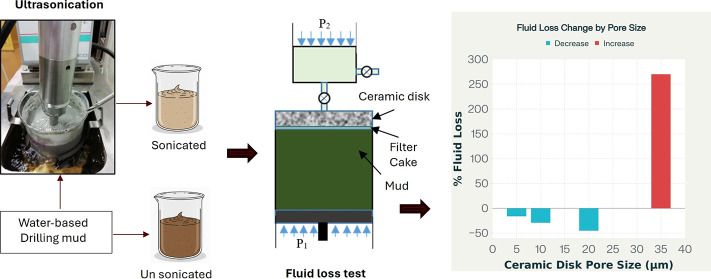

## Introduction

During any conventional oil and gas drilling project, the properties of the drilling fluids are highly important for ensuring safe, efficient, and reliable operations. Drilling mud, which is typically a carefully formulated mixture of water (or oil), clay, and other additives, including weighting agents, is pumped into the wellbore under high pressure to ensure that the borehole remains filled with mud during drilling operations. Drilling mud serves several critical functions, including providing cooling and lubrication for the drill bit, minimizing wear and tear, maintaining hydrostatic pressure, preventing kicks, removing rock cuttings and other debris generated during drilling from the borehole, and preventing borehole collapse and thereby ensuring the integrity of the drilling operation.^1^ Careful selection of the drilling fluid is therefore important to reduce costs, minimize the impact on the environment, and improve the efficiency of well drilling.^2^

In most drilling operations, drilling mud is generally pumped into the wellbore at pressures higher than the rock formation pressure. However, in such cases, the fluids from the mud can invade the reservoir, a phenomenon commonly referred to as filtrate or fluid loss.^1^ This filtrate, which invades the formation, can displace existing formation fluids and create a damage zone around the wellbore, which can impair the well’s productivity. Hence, proper formulation and testing of the drilling fluid are necessary to minimize the amount of filtrate entering the formation. To achieve this, fluid loss control additives are usually added to drilling mud, which can be either chemical-based additives or novel nanoparticle additives such as carbon nanotubes (CNTs).^1^

The solid phase component, typically bentonite in drilling mud, is preferentially deposited on the borehole wall, creating a filter cake layer (mud cake). A good filter cake can reduce filtrate loss from the drilling mud by forming a thin, low-permeability layer that limits additional fluid loss into the formation. The permeability of the filter cake regulates the flow of the fluid into the formation. However, an unchecked accumulation, leading to a thicker filter cake, may lead to an increase in the circulating pressure that can irreparably damage the borehole. Therefore, for an effective drilling procedure, controlling the filter cake thickness and fluid loss properties is essential.^3^

Many studies have examined filtrate loss into formations, its damaging effects, and the effectiveness of loss control additives in reducing this issue.^4,5^ Several commercial loss control additives have been developed by industry to reduce filtrate loss, especially natural and synthetic polymer-based additives such as carboxy-methyl cellulose (CMC), polyanionic cellulose (PAC), cyclodextrin, and epichlorohydrin, among others.^6^ Other loss control agents, such as Resinex, hydroxyethyl cellulose (HEC), calcium carbonate (CaCO_3_), xanthan gum, acrylamide (AAM), and the cross-linker polyethylenimine, have also been used as loss control agents with varying degrees of success.^7,8^ However, every drilling operation necessitates the large-scale addition of these chemicals, which are not always economically feasible and can occasionally be harmful to the environment and human health.^9^

Studies by Mahmoud et al.,^3^ Nasser et al.,^11^ Martin et al.,^12^ Salih et al.,^9^ Ghasemi et al.,^13^ Kanjirakat et al.^14^ have explored the impact of adding nanomaterials such as SiO_2_ and CNTs to drilling mud to reduce filtration loss in boreholes. These studies were based on the concept that the interaction between the nanoparticles and the active components in the mud can lead to agglomeration (flocculation), thereby resulting in reduced fluid loss.^4,14,15^ The tiny nanomaterials also act as bridging agents, plugging the pores in the mud cake and thereby preventing fluid loss into the formation.^16^ However, challenges and concerns remain over the use of nanomaterials due to the potential for reduced cutting and debris-carrying capacity of the drilling mud, leading to poor hole cleaning.^17^

The use of mechanical energy to alter the mud characteristics and rheology of drilling mud has been suggested as an alternative to conventional chemicals; however, work on this topic has been limited. Research by Rana et al.^18^ looked into the ultrasonic dispersion of nanoparticles in fluid media and suggested that it could be used to prepare drilling muds that are highly uniform and without agglomeration of clay particles in the liquid medium, thereby improving fluid retention characteristics to prevent shale swelling. Vakilinia^19^ investigated the use of ultrasonic wave radiation at optimized time intervals during wellbore stimulation to reduce mud filtration into porous media and reduce the formation of mud cakes. Exposing the porous media to ultrasonic waves for 10–30 s was reported to reduce the damage zone around the wellbore while simultaneously improving permeability. Majidi et al.^20^ investigated the effect of changing the rheology of drilling mud on reducing fluid loss and noted that shear thinning of the fluid increased mud losses, whereas yield stress controlled the overall volume of fluid lost into the formation. Work by Ahmad et al.^21^ and Ruan and Jacobi^22^ also reported that ultrasonication of nanoparticle-laden drilling mud improved its colloidal stability and reduced its aggregation tendency, thereby resulting in a more uniform mud cake and reduced filtrate loss. Apart from mixing and creating a uniform dispersion of colloids in drilling fluids,^23^ high-energy ultrasonic waves have also typically been used in industry for several years to solve issues related to near-wellbore formation damage,^24^ hole cleaning, formation imaging, improved oil recovery, and methods for removing unwanted suspended particles in various fluid mixtures.^25^ When evaluating the use of ultrasonication to improve the properties of drilling mud, much of the work has focused only on novel formulations and blends with nanoparticles, the properties of which have been optimized via standard sonication methods to improve rheological properties and reduce filtrate loss and mud cake thickness.^26–29^ While positive effects of ultrasonication were noted on the combined mud–nanoparticle fluid system in these studies, no efforts were made to document the isolated effect of sonication on only the drilling mud.

Very few studies in the literature have evaluated the impact of ultrasonication on base drilling mud fluid loss characteristics, especially with respect to the filtrate loss and mud cake thickness. Work performed by Guo et al.^30,31^ used water-based mud (WBM) with sulfonated wood coal (SMC) and vinyl copolymer (VCP) as fluid loss additives and systematically evaluated the impact of ultrasonication on the API filtrate loss characteristics and mud cake thickness. Their work demonstrated that ultrasonication of drilling mud created stable mud mixtures with reduced filtration loss as well as thin but dense filter cakes, both of which are beneficial in current drilling applications. However, the study by Guo et al. did not analyze filtration through different pore sizes. Huang et al.^32^ evaluated rheological changes in water-based muds when ultrasonicated. A WBM with calcium bentonite as the base was used for these tests, and the viscosity, yield point, and filtration loss were tested before and after ultrasonication. This study revealed that the filtration loss increased immediately after sonication, but after the sample was aged for a period of time, the filtrate loss decreased significantly. Similar results were also noted in the work of Peng et al.^33^ on sulfonated drilling fluids. They noted that increasing the ultrasonication time and intensity resulted in thinner and denser mud cakes, while the filtrate loss was reduced on average by at least 24.7%.

However, a key limitation of these studies was that most of them were performed on specialized mud formulations (sulfonated/calcium bentonite) or when specific filtrate loss reducers (SMC or very small copolymer) were used, and the results were not compared with those of base mud before and after ultrasonication. Moreover, the influence of filtrate loss and mud cake formation as drilling mud traverses various pore sizes in both sonicated and unsonicated samples has not been reported. The amount of fluid loss during the drilling process is predominantly influenced by the way in which solid particles within the drilling mud obstruct the pores of the rock formations. When these particles accumulate and clog the pores, they significantly restrict the permeability of the rock, thereby affecting the flow of fluid.^29^ By strategically modifying the size and distribution of these solid particles in drilling mud, it is possible to regulate and enhance the filtration properties of the mud effectively without the use of any additional additives. This deliberate adjustment enables better control over fluid loss, ultimately optimizing drilling operations.

Additionally, in studies in which nanoparticles are used as fluid loss control agents, ultrasonication is generally used to disperse these nanoparticles within drilling mud. Although the effects of nanoparticles on fluid loss characteristics are noted in those studies, the less visible or indirect consequence of ultrasonication on altering the sizes of mud solid particles and the consequent effect on fluid loss characteristics have not been addressed thus far. Most studies emphasize the impact of new additives while overlooking the additional effects of the ultrasonication mixing method used to incorporate the components into the mud. This study focuses specifically on evaluating the impact of ultrasonication on the filtrate loss properties of a common water-based mud without the addition of any specific fluid loss reducer or specialized bentonite formulations. Unlike the previous works cited in the literature, this work is unique since no additional filtrate loss solids are added to the drilling mud, and the effect of ultrasonication alone is investigated for fluid loss. By isolation of the additives from this analysis, the research highlights the specific impact of ultrasonication on fluid loss. The key areas of focus in this study include the effects of ultrasonication on particle size distribution, filter cake thickness, and the resulting fluid loss into the rock formation. If a fluid loss reduction is achieved, this methodology should be highly cost-effective and safe to implement in comparison with conventional methodologies. To experimentally quantify the effect of ultrasonication on drilling mud, a standard fluid loss experiment is performed on sonicated and unsonicated drilling mud samples as they pass through ceramic disks with pore throat sizes of 5, 10, 20, and 35 μm, which mimic the porous structure of rock formations.

## Experimental Procedure

The primary phase of the experimental work involved formulating the drilling mud sample needed for all planned tests. This study analyzes both ultrasonicated and unsonicated drilling mud samples. The sonicated mud samples were prepared by ultrasonication for 60 min at frequencies exceeding 20 kHz. A probe-type ultrasonicator (Hielscher UIP1500hd) was utilized for this process. Following the preparation of the samples, fluid loss tests were conducted in accordance with the practices recommended by the American Petroleum Institute (API) RP 13I. A simplified schematic illustrating the methodology used for the experimental evaluation is presented in Figure 1.

**Figure 1:**
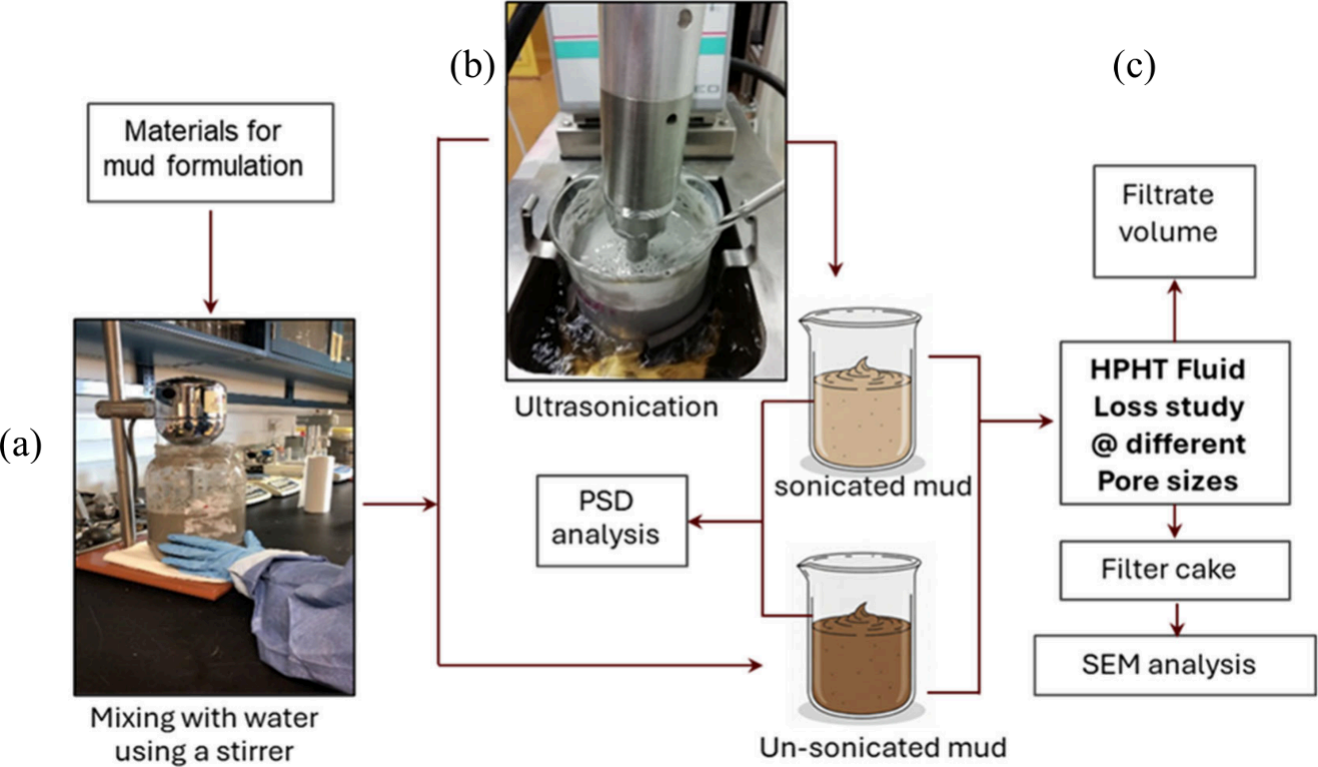
Schematic of the methodology used in the study. (a) Preparation of drilling mud, (b) ultrasonication of mud, and (c) HPHT fluid loss and characterization studies.

### Drilling Mud and Sample Preparation

A drilling mud formulation with a mud weight (density) of 12.0 ppg (pounds per gallon; 1.44 g/cm^3^) was prepared, as represented in Table 1. This mud sample was used as the base for comparing all experimental investigations into the effects of ultrasonication on mud filtrate loss characteristics and filter cake properties. The mud preparation began with 266 mL of deionized water (representing the laboratory equivalent of one barrel of drilling fluid), which was poured into a clean mixing cup as the base fluid. To control the water chemistry and neutralize the hardness, 0.3 g of sodium carbonate (Na_2_CO_3_) was added, and the mixture was mixed for 2 min. Then, 0.3 g of sodium hydroxide (NaOH) was introduced to adjust the alkalinity and stabilize the pH of the mixture. The solution was stirred for an additional minute to ensure a uniform pH adjustment. Next, 108 g of sodium chloride (NaCl) was added to increase the salinity, simulate the formation water properties, and control the fluid density. The mixture was stirred until the salt was fully dissolved, as shown in Figure 1a. To modify the rheological properties of the fluid, 1.5 g of a proprietary viscosifier was added, and the mixture was mixed for 7 min. This component enhanced the ability of the fluid to suspend and transport drill cuttings. Next, 5 g of a polymer-based fluid stabilizer was added for additional viscosity control, and the mixture was then blended for 10 min to ensure complete dispersion. Finally, 1.5 g of barite (BaSO_4_) was gradually added as a weighting agent to increase the fluid density, which is essential for downhole pressure control and achieves the desired final mud weight of 12.0 ppg. The final mixture was stirred for an additional 10 min to achieve a uniform composition. The prepared drilling fluid was then used for further laboratory testing, including filtration and rheological performance evaluations, under controlled conditions to assess its field applicability.

**Table 1:** Base Drilling Mud Formulation

Components	Density, g/mL	Mass, g	Volume, mL
Deionized water	1	266.3	266.3
pH buffer - sodium carbonate	2.54	0.3	0.1
pH buffer - sodium hydroxide	2.13	0.3	0.1
Density/temperature control - sodium chloride	2.16	108	50
Viscosity modifier (commercial)	1.5	1.5	1
Polymer-based fluid stabilizer (commercial)	1.5	5	3.3
Weighting agent - barite	4.2	122.5	29.2
Total mass and volume		503.9	350
Mud weight (ppg)		12.00	

Once the base drilling mud was prepared, it was separated into two batches, one subjected to ultrasonic energy via a Hielscher UIP1500h ultrasonic processor and the other left untouched to be tested as the base control sample of unsonicated mud. Ultrasonication was conducted at a constant frequency of 20 kHz with an amplitude of 80%, which was chosen to promote cavitation and effective particle dispersion. Ultrasonication was performed for a period of 60 min. Since intense heating occurs in the sample during ultrasonication, the sample was immersed in the fluid bath of a chiller (Figure 1b). Following ultrasonication, all of the samples were subjected to fluid loss evaluation.

### Fluid Loss Experiments

Under high-pressure and high-temperature (HPHT) conditions, a permeability plugging tester (PPT) from OFI Testing Equipment, Inc. (PPT-171-90), was employed to assess fluid loss, which is commonly termed a filtrate/fluid loss test. API Recommended Practice 13I^34^ (API RP 13I), a standard published by the American Petroleum Institute (API), provides procedures for the laboratory testing of drilling fluids where the fluid loss test is conducted at 100 psi and at ambient temperature. However, for the current HPHT fluid loss test, experiments were performed at higher differential pressures and temperatures of 1200 psi and 250 °C. In the HPHT setup design, the filtrate does not drip directly into an open cylinder, as it is too hot. Instead, it passes into a back-pressure receiver, which is itself pressurized to prevent flashing/boiling of the filtrate at high temperature (Figure 2a).

**Figure 2:**
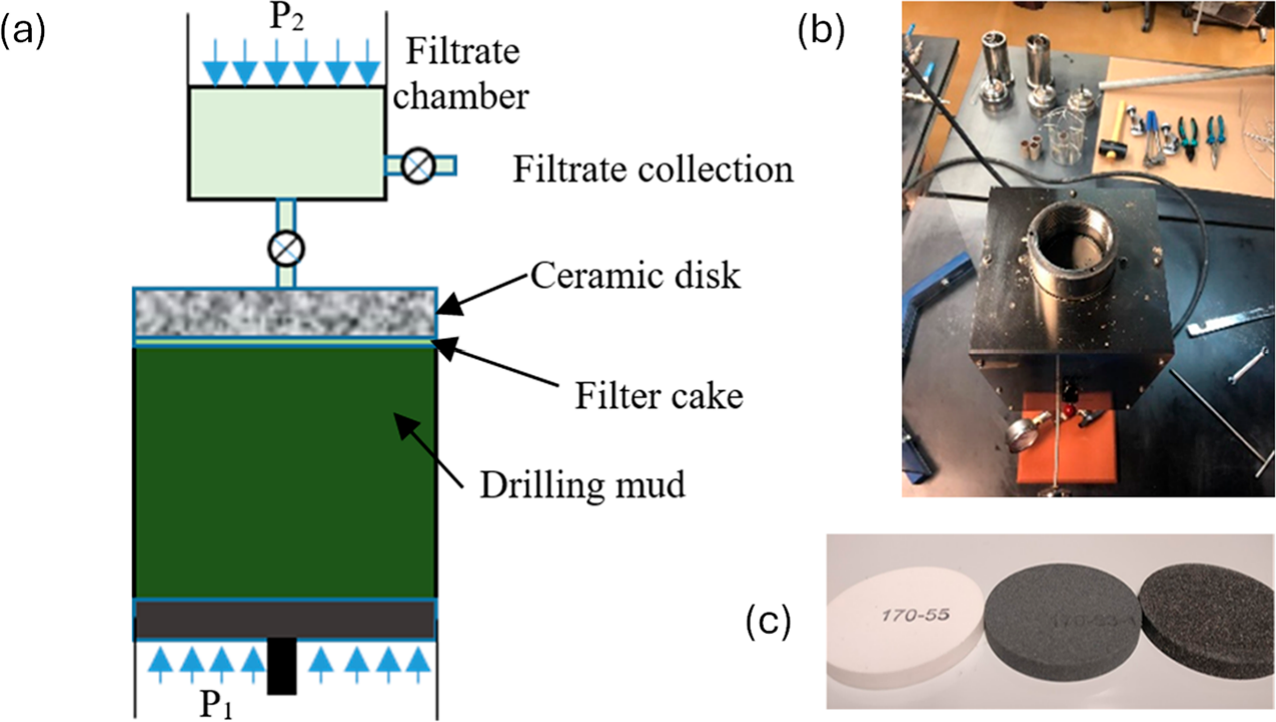
(a) Schematic representation of the HPHT fluid loss experiment. (b) Top view of the HPHT fluid loss experiment setup. (c) Picture of the ceramic disks used (left to right: 5, 10, and 20 μm pore throat disks).

Filtrate loss measurements were conducted to evaluate the filtration performance of the prepared drilling fluid, both before and after ultrasonication. Tests were performed using ceramic filter disks with pore sizes of 5, 10, 20, and 35 μm. Prior to testing, the heating jacket was preheated to 250 °C, while the ceramic disks (Figure 2c) were soaked in the base drilling mud for 10 min to ensure proper saturation. The test cell, as shown in Figure 2b, was assembled with the inlet cap tightened and positioned in the heating jacket, with the inlet side facing downward. The piston was installed, and pressure was gradually applied to properly seat it in the cell. The cell was then filled with the drilling fluid sample to be tested (sonicated or unsonicated mud). The ceramic disk of the required pore size was placed on top, followed by the outlet cap and an outlet valve. A back-pressure receiver (kept at 100 psi) was connected to maintain pressure equilibrium via a CO_2_ cylinder assembly. Once the test temperature (250 °C) was reached, 1300 psi of pressure was applied, and the outlet valve was opened to initiate filtration with a differential pressure of 1200 psi.

Even though the test is normally run for 30 min and the 30 min cumulative filtrate is the usual reported filtrate loss value, it is a common lab practice to record the cumulative collection at 30 s and 1, 5, 7.5, 15, and 30 min. Once the test was completed (e.g., after 30 min), the receiver was cooled, the pressure was safely released, and the total volume of filtrate was measured.

### Filter Cake Analysis

Ceramic disks were used in the filtrate loss tests to replicate porosities similar to those found in rock formations. These disks are typically classified according to their mean pore throat sizes, and ceramic disks with mean pore sizes of 5, 10, 20, and 35 μm were used in the study. By changing the porosity of the filter disks, we studied the effect of the pore size on the filtration characteristics of the drilling fluids.

During the fluid loss experiment, the drilling mud fluid passed through a porous disk, leading to the formation of a filter cake at the bottom of the ceramic disk. After each fluid loss test, the ceramic disk was removed from the PPT cell along with the filter cake. The filter cake was gently rinsed with water to remove the excess fluid. The thickness of the filter cake was measured via a precision Vernier caliper (±0.02 mm) at three different locations. The thickness measurement was recorded for reporting, and the visual quality of the filter cake was noted for each scenario.

The morphology of the particulates and the surface characteristics of the unused ceramic disks were then studied by scanning electron microscopy imaging (Thermo Fisher Apero SEM). In addition, a laser diffraction particle size analyzer (Beckman Coulter, LS13320) was used to quantify the particle size distribution of the solid components of the drilling mud sample.

## Results and Discussion

The test scenarios for evaluating the effects of sonication on drilling mud are summarized in Table 2. Both sonicated and unsonicated (base) mud samples were tested using ceramic disks with pore sizes of 5, 10, 20, and 35 μm. The resulting filtrate volume and filter cake properties were analyzed. Additionally, particle size distribution analysis and scanning electron microscopy (SEM) imaging of the unused ceramic disks were performed to qualitatively analyze the interaction between the particles and pore sizes and their impact on the fluid loss characteristics of sonicated and unsonicated mud samples.

**Table 2:** Evaluation Criteria for Sonicated and Unsonicated Muds

Parameters	Range
Fluid loss	Cumulative filtrate volume at 30 s, 1 min, 5 min, 7.5 min, 15 min, and 30 min
Ceramic disk pore throat size	5, 10, 20, and 35 μm
Filter cake analysis	Visual, average thickness (mm)
Mud particle size distribution	For 60 min of sonication
Mud particle morphology	Scanning electron microscopy (SEM)
Pore size distribution of ceramic disks	SEM of unused 5, 10, 20, and 35 μm disks

### (i) Fluid Loss Studies with Different Pore Size Ceramic Disks

Figure 3 shows the amount of filtrate volume collected from sonicated and unsonicated drilling mud samples as they passed through a 5 μm pore throat size disk. The volumes collected at different time intervals are shown. Both sonicated and unsonicated drilling mud samples exhibit a nonlinear fluid loss pattern over time, with comparable trends observed in both sample types. Up to the 10 min interval, the filtrate volumes from the sonicated and unsonicated mud samples were nearly identical. However, a noticeable difference in total filtrate volume emerged at 30 min intervals. Furthermore, although the difference is marginal, fluid loss is consistently greater in the unsonicated mud than in its sonicated counterpart. After 30 min, the sonicated drilling mud helped reduce fluid loss by at least 16%.

**Figure 3:**
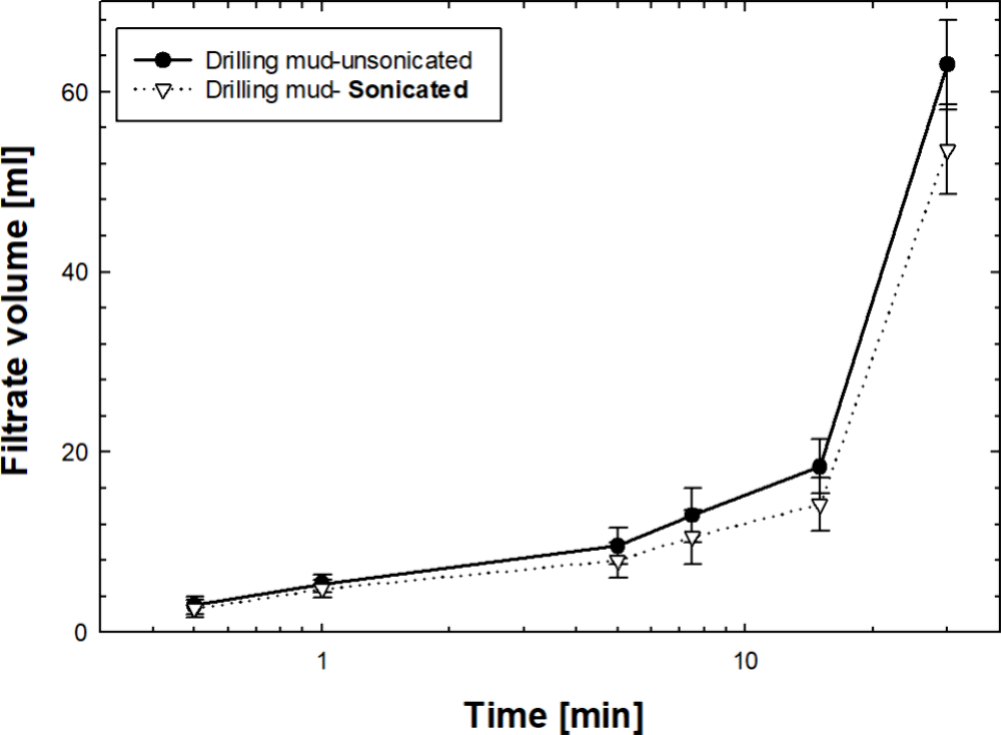
Filtrate volume collected for sonicated and unsonicated drilling mud at different time intervals while passing through a 5 μm pore size ceramic disk.

Figure 4 shows the volume of filtrate collected from a disk with a pore throat size of 10 μm. In contrast to the flow through a disk with a pore throat size of 5 μm, there was a substantial difference in the amount of filtrate collected, even during the initial stages of sample collection. Notably, there was a significant difference in the volume of filtrate collected after just 1 min. This trend persists at longer time intervals, as well. These findings indicate that fluid loss is reduced when sonicated mud with a 10 μm pore throat size disk is used. Moreover, the total filtrate volume obtained from the sonicated mud was at least 29% lower than that derived from unsonicated mud.

**Figure 4:**
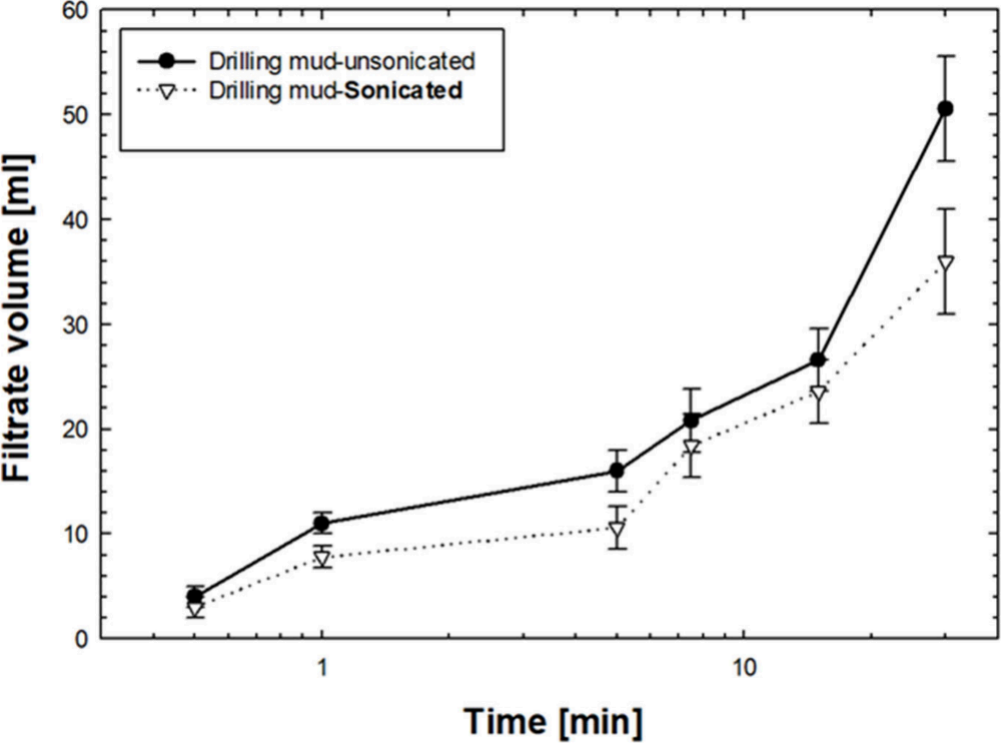
Filtrate volume collected for sonicated and unsonicated drilling mud at different time intervals while passing through a 10 μm pore size ceramic disk.

Figure 5 presents the volume of the filtrate collected during the passage of samples through a disk with a pore throat size of 20 μm. The trend in fluid loss is similar to that observed for a disk with a pore throat size of 10 μm. A significant reduction of approximately 45% in the amount of filtrate is noted when sonicated drilling mud is employed in contrast to unsonicated mud. This decrease in fluid loss has important implications for reducing drilling costs in practical applications.

**Figure 5:**
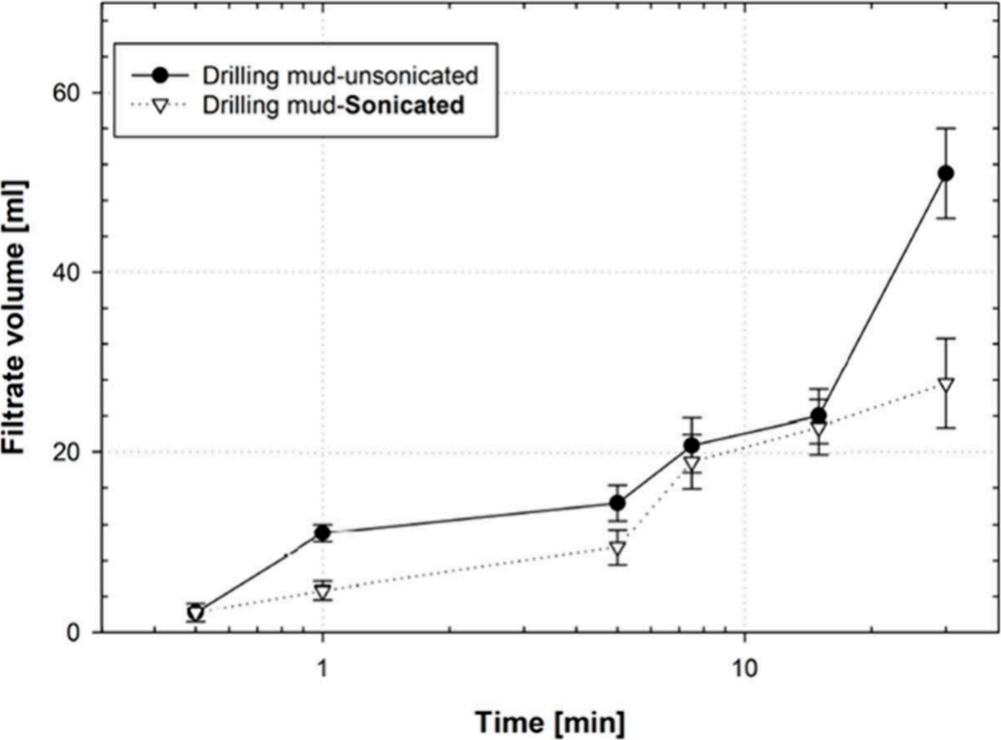
Filtrate volume collected for sonicated and unsonicated drilling mud at different time intervals while passing through a 20 μm pore size ceramic disk.

Figure 6 displays the filtrate volume collection data for both sonicated and unsonicated drilling mud samples during a fluid loss test conducted on a ceramic disk with an average pore throat size of 35 μm. In contrast to previous observations, the sonicated drilling mud samples presented a significant increase (270%) in the level of fluid loss. In comparison, the fluid loss value for the unsonicated mud is similar to that observed with a disk having a pore throat size of 20 μm. These findings indicate that the fluid loss characteristics of the unsonicated drilling mud samples remained consistent, even when they passed through ceramic disks with pore throat sizes ranging from 5 to 35 μm. However, the fluid loss trends for sonicated drilling mud samples were strongly affected by the average pore throat sizes of the ceramic disks. The consolidated observations of the cumulative volume of filtrate collected for the fluid loss study are given in Table 3.

**Figure 6:**
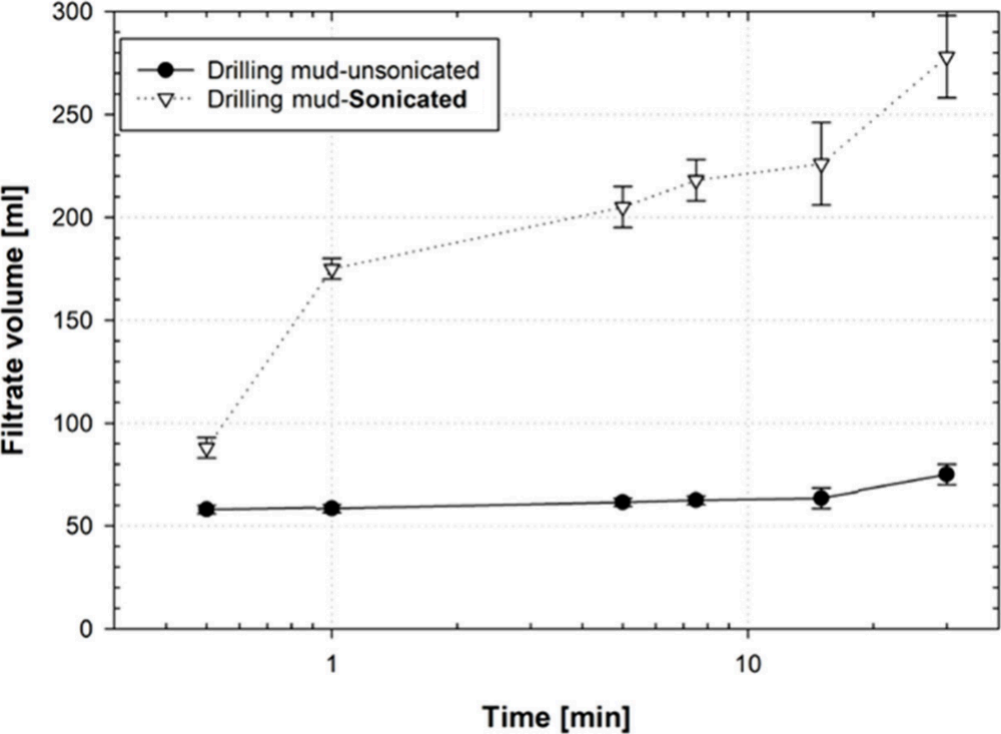
Filtrate volume collected for sonicated and unsonicated drilling mud at different time intervals while passing through a 35 μm pore size ceramic disk.

**Table 3:** Percentage Change in Filtrate Loss in Sonicated and Unsonicated Mud When Passed through Different Pore Throat Sizes

Ceramic Disk Pore Throat Size	Sonicated/Unsonicated % Fluid Loss Change	Type of Change	Sonicated Particle Size
5 μm	16%	Decrease	20 μm
10 μm	29%	Decrease	20 μm
20 μm	45%	Decrease	20 μm
35 μm	270%	Increase	20 μm

Next, the filter cake quality and thickness are analyzed. The average value of the filter cake thickness obtained during the experiments is plotted in Figure 7. The filter cake formed by sonicated mud samples is thinner than that formed by a sample using unsonicated mud. The average thickness of the sonicated mud filter cakes ranged from 0.2 to 0.4 mm, whereas that of the unsonicated mud was twice as thick, ranging from 0.7 to 1 mm. While the thickness of the filter cake alone is not an indicator of better filtrate reduction characteristics, the accompanying fluid loss results, as noted in Table 3, indicate that the sonicated mud filter cakes demonstrate much better filtrate reduction characteristics because of the formation of a thinner and impervious filter cake while significantly reducing fluid loss.

**Figure 7:**
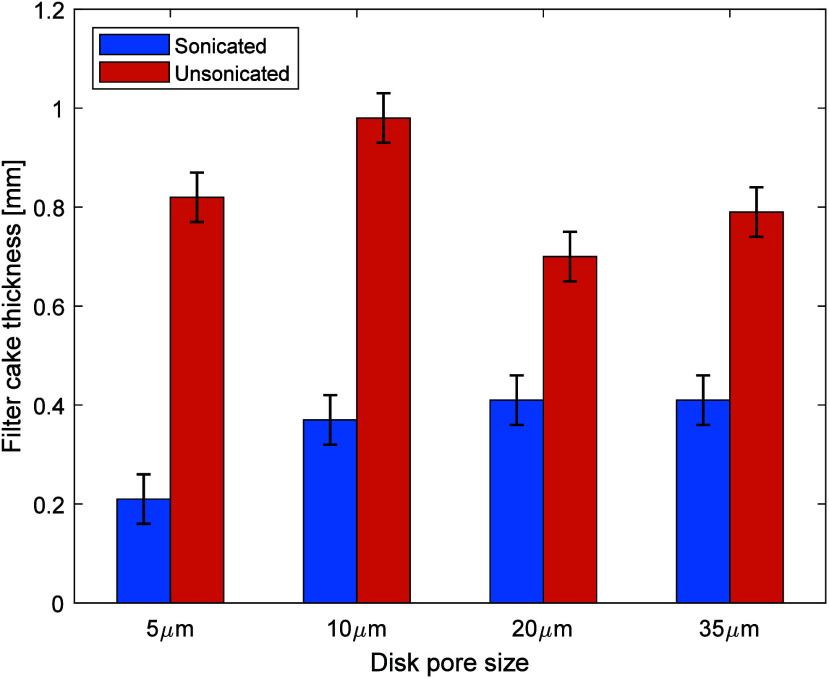
Filter cake thickness measured for sonicated and unsonicated drilling mud samples after fluid loss experimentation with different filter disk pore sizes.

The surface characteristics of the filter cake formed on the ceramic disk were visually analyzed. Figure 8 shows photographs of the filter cakes extracted after experimentation via sonicated and unsonicated drilling mud samples. The filter cakes formed by sonicated mud on 5, 10, and 20 μm pore size disks are thin and uniform. In contrast, the filter cake formed on the 35 μm disk using sonicated mud is thicker, more porous, and exhibits cracks. It appears that the filter cake formed is primarily due to the deposits of larger particles, part of which seems to have broken out while the filter cake was extracted from the test rig. Compared with the sonicated mud sample, the unsonicated drilling mud produced a slightly thicker and nonuniform filter cake.

**Figure 8:**
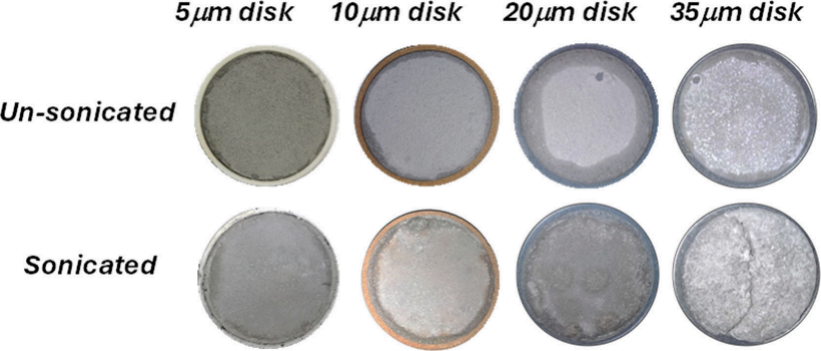
Photographs of the filter cakes extracted after experimentation.

One possible explanation for the reversal in the fluid loss trend observed in the sonicated drilling mud as it passes through a filtration disk with a pore size of 35 μm is related to the effects of the sonication process on the particle size distribution of the solid components of the mud. During sonication, high-frequency sound waves are applied, which can lead to the fragmentation and dispersion of larger particles in the drilling mud. As a result, the average size of these solid particles may be significantly reduced, leading to a shift in their distribution toward smaller sizes. If many newly generated particles are smaller than the pore size of the disk, they will be able to pass through the porous structure more easily. Instead of being trapped by the disk, these fragmented particles could flow through, thus diminishing the overall retention observed with larger particles. Therefore, the interplay between sonication and pore size may be a critical factor in understanding the filtration dynamics of sonicated drilling mud. These facts are analyzed in the next section.

### (ii) Interplay of Mud Particle Size and Ceramic Disk Pore Size

To analyze the effects of ultrasonication on the particle sizes of the solid constituents in drilling mud, the particle sizes were assessed for different durations of sonication. The samples were collected at 15, 30, 40, and 60 min from the start of sonication, and the particle size distribution was studied (Figure 9). As the sonication time increased, the particle size distribution shifted to the left. The unsonicated mud sample shows a distribution peak in the 20–40 μm size range. During ultrasonication, the intense cavitation and shear forces generated by collapsing ultrasound-induced bubbles fragment the agglomerates of barite particles in the drilling mud into finer particles.^35^ Hence, a slight reduction in the mean particle size is observed after 15 min. The ultrasonication process also cleans particle surfaces, improving the wettability of the dispersion,^36^ which further improves the suspension stability of barite in the mud. This ultimately results in the formation of a denser but thinner filter cake. Notably, prolonged sonication can also induce reagglomeration of solid particles in the mud, caused by the high surface energy associated with the very fine particles generated during extended exposure.^37^ After 30 min of ultrasonication, some reagglomeration is evident as the particle size distribution curve flattens, indicating that both smaller and larger particles are formed due to fragmentation and reagglomeration. By 60 min, the peak shifted toward the 20 μm range with an increased concentration of smaller particles; however, reagglomeration also occurred, leading to the presence of particles larger than 100 μm. Nevertheless, sonication consistently reduced the mean particle size with increasing sonication time, yielding a mean size of 20 μm after 60 min of sonication.

**Figure 9:**
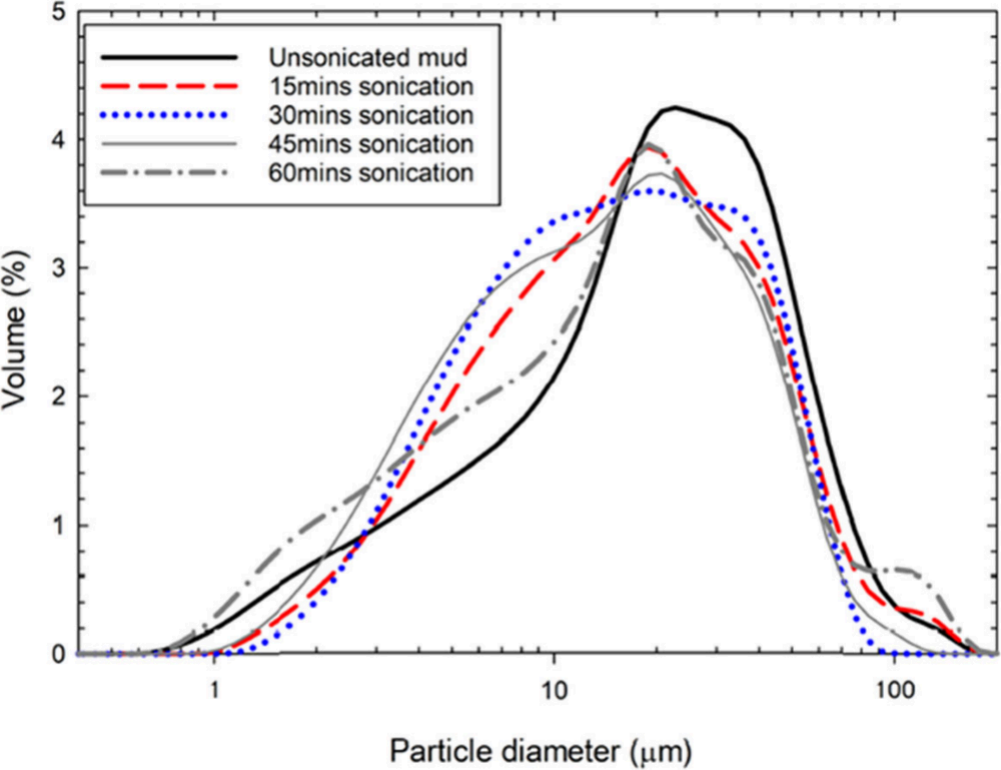
Particle size distribution with varying durations of ultrasonication applied to the drilling mud sample.

Scanning electron microscopy images of both sonicated and unsonicated mud samples were collected. For this purpose, sonicated and unsonicated mud samples were dried on carbon tape, which were sputter-coated before imaging. Figure 10 shows SEM images of the unsonicated and sonicated mud samples, which reveal substantial differences in size and morphology between these samples. The unsonicated sample is characterized by larger, angular, and block-like particles with well-defined edges. In contrast, the sample subjected to 60 min of sonication has a pronounced reduction in particle size and a transformation in morphology, with the particles appearing much finer and forming a denser aggregate. The ultrasonication technique effectively disrupted the larger particles, leading to fragmentation into smaller and more uniform sizes.

**Figure 10:**
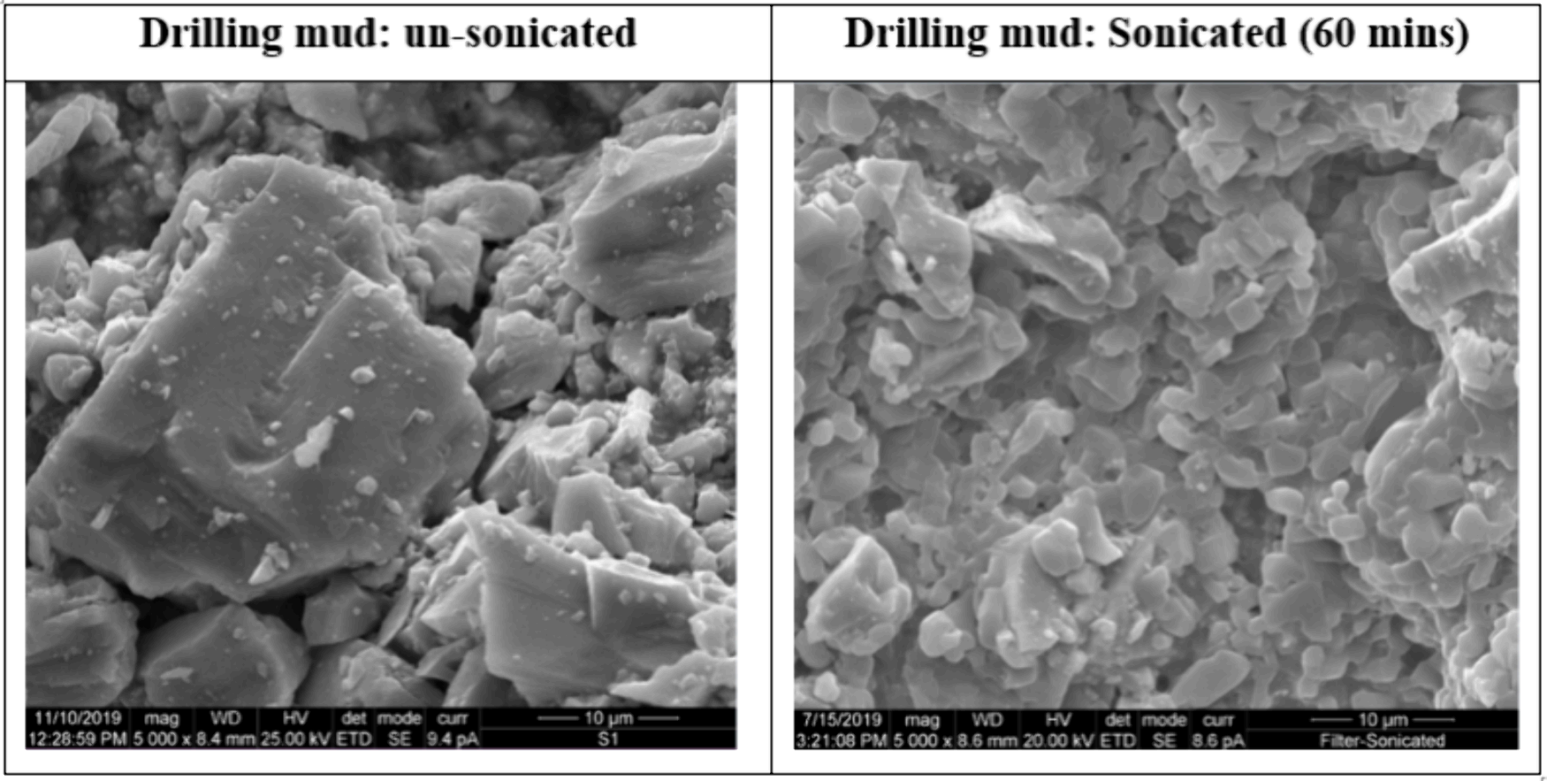
Scanning electron microscopy (SEM) images of unsonicated and sonicated drilling mud samples.

The fluid loss through the ceramic disk is influenced by the relationship between the particle size of the solid components within the drilling mud and the pore size of the ceramic disk through which the fluid is transmitted; thus, determining the pore size is essential. In Figure 11, the actual dimensions of the pores on the unused disk surfaces, captured via a scanning electron microscope, are presented. The pores appear as dark cavities within the SEM images. The shapes of the pores are complex and nonspherical. Given this intricate morphology, the probability of solid particles becoming trapped within pores is significantly high. Only smaller particles are more likely to traverse the elaborate pore structures. For the disk featuring a 5 μm pore throat, the dimensions of the pore openings are primarily observed to range from 4 to 6 μm. This relatively narrow size indicates that only very small particles can effectively pass through these openings. In contrast, the disk with a 35 μm pore throat presents a much broader range of pore openings, varying from 30 to 70 μm. This significant increase in the pore size allows a wider variety of particle sizes to traverse the pore networks. Consequently, smaller particles are considerably more likely to pass through the 35 μm disk than the 5 μm disk.

**Figure 11:**
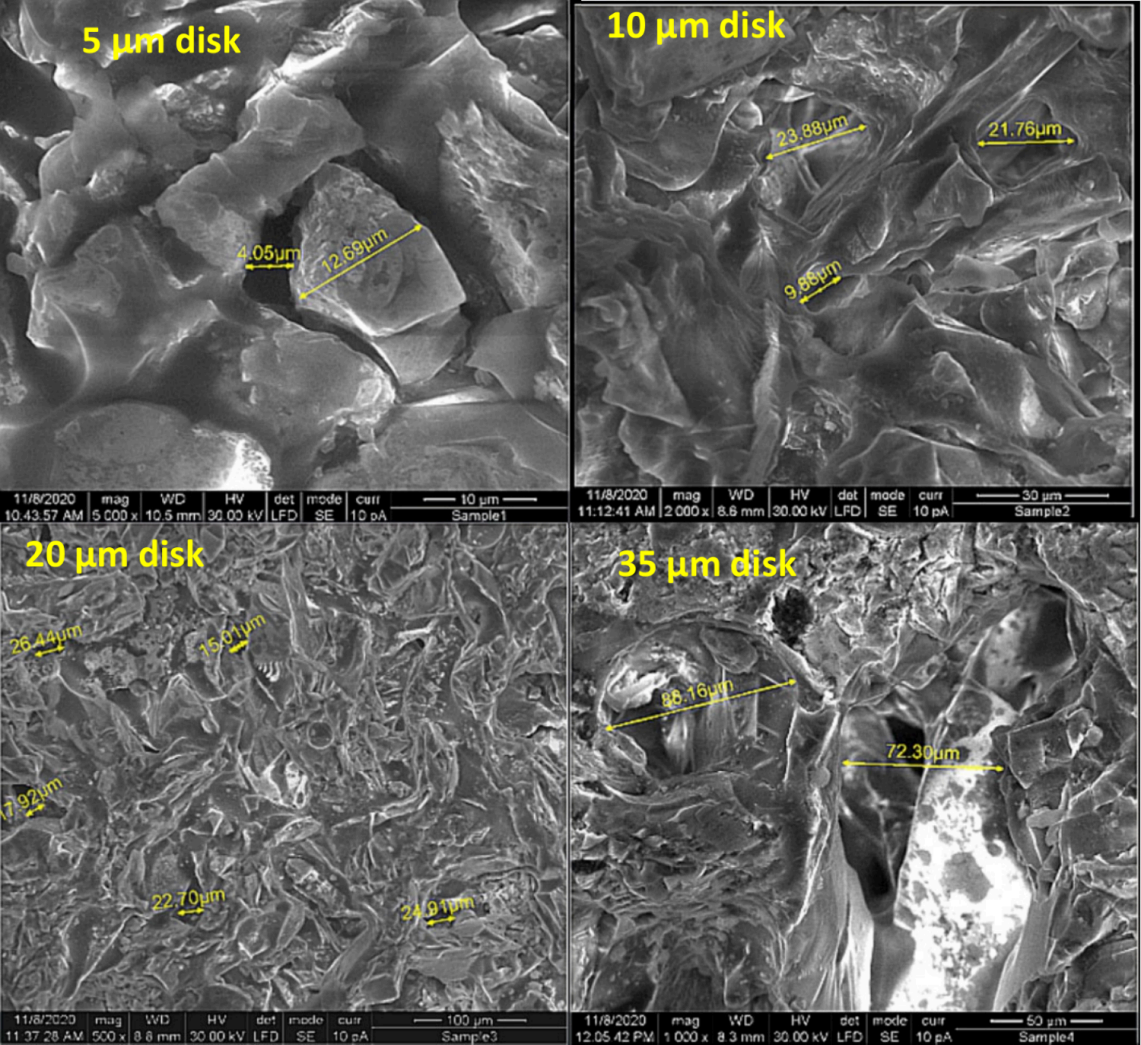
Scanning electron microscopy (SEM) images of the unused ceramic disk surfaces.

SEM images of the particles and pore voids within the disk, along with the provided particle size distribution (Figures 9–11), reveal that both the particle size and the pore size significantly influence the retention of solid particles in the filter disk. The effectiveness of retention highly depends on the interplay between these two factors. The dynamics of particle migration through porous media are significantly influenced by the relative sizes of the pores and the solid particles in the drilling mud. When smaller particles are trapped in material pores, they create a thin filter cake that is less permeable, reducing fluid loss. This phenomenon has been observed in disks measuring 5 to 20 μm. Conversely, larger pores, such as those with a pore throat size of 35 μm, can lead to a more porous filter cake, which increases fluid loss because larger voids allow fluids to escape more easily.

The argument that particle trapping within pore cavities is influenced by the relationship between the average pore size and the sizes of the particles can be better understood through a simple particle retention model. This model uses a probabilistic approach to illustrate how particles are retained by a porous ceramic filter by utilizing a number-weighted particle size distribution. Here, we assume a triangular distribution of particle sizes ranging from 1 to 100 μm with a mode of 20 μm. This simple distribution realistically skews toward finer particles. In the analysis, variations in pore sizes are not considered. The pore throats are treated as absolute cutoffs where the particles with diameters equal to or greater than the throat size are considered retained, whereas smaller particles are assumed to pass through. As described by Chan and Govindaraju,^38^ the probability of retention corresponds to the fraction of the cumulative particle size distribution above the pore throat diameter, which is expressed as

1 $$R\left(d_{p}\right)=1-F\left(d_{p}\right)$$

Assuming a triangular distribution for particle size, the cumulative distribution function is defined as

2 $$F\left(d_{p}\right)=\frac{{\left(d_{p}-a\right)}^{2}}{\left(b-a\right)\left(c-a\right)}\;\mathrm{for}\;a<d_{p}<c$$

3 $$F\left(d_{p}\right)=1-\frac{{\left(b-d_{p}\right)}^{2}}{\left(b-a\right)\left(b-c\right)}\;\mathrm{for}\;c<d_{p}<b$$

where *a*, *b*, and *c* are the lower limit (1 μm), upper limit (100 μm), and mode (20 μm) of the distribution, respectively, and *d*_*p*_ represents the pore throat size.^38^ When the throat size was varied from 5 μm to 10, 20, and 35 μm, the retention probabilities were 99.1%, 95.7%, 80.8%, and 53.4%, respectively. The retention probability remains above 80% for pore sizes of up to 20 μm. However, when a 35 μm pore size disk is used, the retention probability decreases to nearly 50%. This indicates that the smaller particles formed by ultrasonication are likely not retained by the disk, allowing larger particles to dominate the drilling mud, which aids in the formation of the filter cake. Consequently, the filter cake that forms tends to be more porous, leading to an increase in fluid loss.

From the above discussions, it is clear that when ultrasonication is employed on drilling mud to reduce fluid loss, understanding the pore sizes of the rock formations being drilled is essential. The effectiveness of utilizing ultrasonication as a fluid loss control method depends on these rock and fluid characteristics, making it essential to evaluate both the particle size distribution and the pore structure to optimize drilling operations. In addition, when ultrasonication is used as a dispersion technique, especially for nanoparticles in drilling mud, one cannot neglect the effect caused by ultrasonication in modifying the mud particle sizes. While previous studies have reported a reduction in fluid loss primarily due to the interaction of nanoparticles, the less obvious impact of the reduction in particle size caused by ultrasonication has often been overlooked. This reduction in fluid loss may actually be attributed to both the decrease in solid particle size and the effects of the nanoparticles. This study aims to determine whether ultrasonication alone can change the fluid loss characteristics of drilling mud.

## Conclusions

Traditional filtrate loss mitigation approaches rely heavily on chemical additives and nanoparticles, which may alter the mud composition and introduce environmental and cost concerns. As seen in the literature, ultrasonication is frequently employed to disperse nanoparticles in drilling mud, and the use of these nanoparticles has been shown to reduce fluid loss characteristics. However, the indirect effect of particle size reduction caused by ultrasonication and its impact on fluid loss have been overlooked. This study aims to evaluate the effectiveness of ultrasonication alone on the filtration behavior. A combination of filtrate loss tests, particle size analysis, and scanning electron microscopy was used to obtain a clear picture of how sonication modified the base mud properties and how these changes influenced the filtrate loss performance when the mud was passed through different pore sizes ranging from 5 to 35 μm.

The results showed that ultrasonication consistently minimizes fluid loss compared with that of unsonicated samples, particularly those with pore sizes of 5 and 10 μm, which achieve reductions of approximately 16% and 29%, respectively. Notably, even at a larger pore size of 20 μm, a substantial reduction in the filtrate volume of approximately 45% was observed. These improvements were accompanied by the formation of thinner, less permeable filter cakes (0.2–0.4 mm thick) compared to the mud cakes from unsonicated mud (0.7–1 mm thick). Scanning electron microscopy and particle size distribution data confirmed that ultrasonication fragmented larger particles and dispersed mud aggregates into finer, more uniform solids, improving suspension stability and promoting the formation of denser, more effective filter cakes. These improvements demonstrate the potential of sonication to enhance the performance of drilling mud in low- to medium-permeability formations.

However, the behavior of sonicated drilling mud changed when it passed through a 35 μm pore size disk, illustrating a significant increase (270%) in the fluid loss. This variation suggests that the effectiveness of ultrasonication depends on the particle size distribution within the mud, with smaller particles bypassing wider pores, resulting in poor retention and cracked filter cakes. SEM imaging of the filter cakes further highlighted the role of pore geometry, with narrow pore throats in the 5 μm disks effectively restricting the passage of particles, whereas the larger 35 μm disk allowed a broader range of particles to pass. The particle retention model used in this study supported these results, showing retention probabilities of over 80% for pore sizes up to 20 μm, while it dropped to approximately 53% for 35 μm pores. This sharp decline explains the poor performance of sonicated mud in the larger pore systems.

Overall, the results of this study demonstrate that simple ultrasonication alone of drilling mud can be highly effective in reducing fluid loss and producing thinner, less permeable filter cakes for small to moderate formation pore throat sizes (≤20 μm). These unintended positive effects of ultrasonication on base drilling mud have not been previously studied, and the results highlight that the success of ultrasonication is formation dependent and that the characterization of pore throat structures is necessary for effective field application. While this study utilizes only a single mud configuration, any future work should investigate filtrate loss with different mud formulations, mud weights, and varying concentrations of mud components. The effects of ultrasonication intensity and duration on rheology, filtrate loss, and mud cake properties, as well as the particle size distribution of drilling mud, also need to be studied. Nevertheless, this novel approach offers an easy, cost-effective, and clean alternative to conventional methods using additives and can help in implementing better mud programs in conventional drilling operations without the need for improved filtrate loss additives.

## Data Availability

All data supporting the findings of this study are available within the article in the form of tables and figures.
